# Dopaminergic tone persistently regulates voltage-gated ion current densities through the D1R-PKA axis, RNA polymerase II transcription, RNAi, mTORC1, and translation

**DOI:** 10.3389/fncel.2014.00039

**Published:** 2014-02-17

**Authors:** Wulf-Dieter C. Krenz, Anna R. Parker, Edmund W. Rodgers, Deborah J. Baro

**Affiliations:** Department of Biology, Georgia State UniversityAtlanta, GA, USA

**Keywords:** stomatogastric, Kv4, HCN, small noncoding RNA, argonaute, conductance ratio, crustacean, activity-dependent

## Abstract

Long-term intrinsic and synaptic plasticity must be coordinated to ensure stability and flexibility in neuronal circuits. Coordination might be achieved through shared transduction components. Dopamine (DA) is a well-established participant in many forms of long-term synaptic plasticity. Recent work indicates that DA is also involved in both activity-dependent and -independent forms of long-term intrinsic plasticity. We previously examined DA-enabled long-term intrinsic plasticity in a single identified neuron. The lateral pyloric (LP) neuron is a component of the pyloric network in the crustacean stomatogastric nervous system (STNS). LP expresses type 1 DA receptors (D1Rs). A 1 h bath application of 5 nM DA followed by washout produced a significant increase in the maximal conductance (*G*_max_) of the LP transient potassium current (*I*_A_) that peaked ~4 h after the start of DA application; furthermore, if a change in neuronal activity accompanied the DA application, then a persistent increase in the LP hyperpolarization activated current (*I*_h_) was also observed. Here, we repeated these experiments with pharmacological and peptide inhibitors to determine the cellular processes and signaling proteins involved. We discovered that the persistent, DA-induced activity-independent (*I*_A_) and activity-dependent (*I*_h_) changes in ionic conductances depended upon many of the same elements that enable long-term synaptic plasticity, including: the D1R-protein kinase A (PKA) axis, RNA polymerase II transcription, RNA interference (RNAi), and mechanistic target of rapamycin (mTOR)-dependent translation. We interpret the data to mean that increasing the tonic DA concentration enhances expression of a microRNA(s) (miRs), resulting in increased cap-dependent translation of an unidentified protein(s).

## Introduction

Dopaminergic systems use volume transmission to modulate cognitive and motor functions (Zoli et al., 1998; Schultz, 2007; Oginsky et al., 2010). Tonic and burst firing neurons release Dopamine (DA) that can then diffuse and act predominantly at remote extra-synaptic receptors before reuptake by DA transporters. As a result, target neurons are tonically exposed to DA; e.g., approximately tens of nM in the striatum and prefrontal cortex (Owesson-White et al., 2012; Nirogi et al., 2013; Zuo et al., 2013), and superimposed upon this baseline are periodic fluctuations in DA that can transiently rise to ~µM levels near the release sites of bursting DA neurons (Park et al., 2011; Rice et al., 2011; Owesson-White et al., 2012).

Phasic and tonic DA have distinct roles in the CNS. Phasic DA may encode reward prediction error (Steinberg et al., 2013), provide sustained motivational drive (Howe et al., 2013) and modulate motor behaviors (Gerfen and Surmeier, 2011). On the other hand, tonic DA is thought to have an enabling function because tonic administration of drugs, such as L-dopa or neuroleptics, can enable motor, motivational and cognitive behaviors (Schultz, 2007). The effects of tonic DA have largely been attributed to D2Rs, but all receptors can show high and low affinity states and there is increasing evidence that tonic DA acting at high affinity type 1 DA receptors (D1Rs) may also enable and shape circuit output over the long-term (Trantham-Davidson et al., 2004; Rodgers et al., 2011a,b; Wall et al., 2011; Saba et al., 2012).

We previously showed that the sole lateral pyloric (LP) neuron in the stomatogastric nervous system (STNS) of the spiny lobster, *Panulirus interruptus*, expressed high and low affinity D1Rs but not D2Rs (Zhang et al., 2010; Rodgers et al., 2011a,b; Krenz et al., 2013). Low affinity LP D1Rs were activated by µM DA to produce immediate and reversible alterations in the biophysical properties of LP voltage gated ionic currents (Harris-Warrick et al., 1995; Johnson et al., 2003; Kloppenburg et al., 2007; Zhang et al., 2010). High affinity LP D1Rs activated by nM DA produced effects over two time scales. They rapidly conferred activity-dependence upon LP *I*_h_ to maintain a conductance ratio and its activity correlate (Krenz et al., 2013), and they also acted through a slower process(es) to persistently influence ion current densities. A 1 h application of 5 nM DA or saline (control) to the superfusate bathing LP, followed by a 4 h washout and subsequent voltage clamp to measure LP *I*_A_ showed that LP *I*_A_ *G*_max_ was significantly increased by 25% in the DA-treated relative to control preparations (Rodgers et al., 2011b). If the experiment was repeated, but LP activity was altered during the 1 h 5 nM DA (or saline) application, then LP *I*_h_ was also significantly increased by 55% in DA-treated preparations relative to saline controls (Rodgers et al., 2011a). Here we examine the cellular processes mediating DA’ s persistent effects and show that many of the same elements involved in long-term synaptic plasticity underpin DA-induced long-term intrinsic plasticity.

## Materials and methods

### Animals

California spiny lobsters, *Panulirus interruptus*, were purchased from Marinus Scientific (Long Beach, CA) and Catalina Offshore Products (San Diego, CA). Lobsters were maintained at 16^°^C in aerated and filtered seawater. Animals were anesthetized on ice before dissection.

### Chemicals and peptides

Tetrodotoxin (TTX), flupenthixol and myristoylated PKI_(14–22)_ were purchased from Tocris Bioscience (Bristol, UK), flavopiridol was from Selleckchem (Houston, TX), and all other chemicals were purchased from Sigma-Aldrich (St. Louis, MI). Peptides were synthesized by Biomatik (Wilmington, DE). DA was made fresh every 30 min to minimize oxidation. In all experiments, antagonists were administered 10 min before DA application. Rp-cAMPS (1 mM) effectively blocks protein kinase A (PKA) in several arthropod models such as *Drosophila* and crustaceans, including *Panulirus* (Erxleben et al., 1995; Kuromi and Kidokoro, 2000; Zhang et al., 2010). PKI is an effective blocker of the PKA catalytic subunit in crustaceans (Dixon and Atwood, 1989). Dosages of rapamycin (100 nM), anisomycin (30 µM) and actinomycin D (50 µM) were previously demonstrated to be effective in several invertebrate models including *Panulirus* (Rodgers et al., 2011a). Concentrations of flavopiridol (100 nM) and 5, 6-dichloro-1-β-D-ribobenzimidazole (DRB, 100 µM) were chosen based on previously demonstrated effective dosages (Chao and Price, 2001; Bensaude, 2011; Yuan and Burrell, 2013).

### Experimental preparation

The STNS was dissected and pinned in a Sylgard lined Petri dish using standard techniques (Selverston et al., 1976). The stomatogastric ganglion (STG) was desheathed and isolated with a Vaseline well. The STG was superfused with saline consisting of (in mM) 479 NaCl, 12.8 KCl, 13.7 CaCl_2_, 39 Na_2_SO_4_, 10 MgSO_4_, 2 glucose, 4.99 HEPES, 5 TES at pH 7.4. Intracellular somatic recordings used to identify neurons were obtained with sharp high resistance glass microelectrodes filled with 3 M KCl (20–30 MΩ) and an Axoclamp 2B amplifier (Axon Instruments, Foster City, CA). Neurons were identified by correlating action potentials from somatic intracellular recordings with extracellularly recorded action potentials on identified motor nerves, and by their characteristic shape and timing of oscillations. The process of dissection and cell identification usually took 3–5 h.

### Somatic two-electrode voltage clamp (TEVC)

For two-electrode voltage clamp (TEVC) of LP *I*_h_, the well surrounding the STG was superfused for 1 h with blocking saline: saline containing 10^−6^ M picrotoxin to block inhibitory glutamatergic synaptic inputs (Marder and Eisen, 1984; Cleland and Selverston, 1995), 10^−7^ M TTX to block voltage-gated Na^+^ channels, 2 × 10^−2^M tetraethylammonium (TEA) to block voltage-gated K^+^ channels, 2 × 10^−4^M cadmium chloride (CdCl_2_) to block Ca^2+^- and Ca^2+^-dependent channels. The LP neuron was next impaled with two low resistance voltage clamp micropipettes (8–10 MΩ when filled with 3 M KCl) connected to Axoclamp 2B or 900A amplifiers (Molecular Devices, Foster City, CA). LP was clamped to a −50 mV holding potential using pClamp software. *I*_h_ was elicited using a series of 4 s hyperpolarizing voltage steps, from −60 mV to −120 mV in 10 mV increments with 6 s between steps. Steady state peak currents were measured by fitting the current trace back to the beginning of the hyperpolarizing voltage step or by subtracting the initial fast leak current from the slowly developing peak of *I*_h_ at the end of each negative voltage step. Currents were converted to conductance (*G* = *I*_peak_/(*V*_m_–*V*_rev_) and fitted to a first order Boltzmann equation. *V*_rev_ *I*_h_ = −35 mV (Kiehn and Harris-Warrick, 1992). For TEVC measurement of LP *I*_A_ the command potential was stepped from −50 mV to −90 mV for 200 ms to remove resting inactivation. The deinactivating prepulse was immediately followed by a 400 ms testpulse to activate the channels. Activation pulses ranged from −40 to +40 mV in 10 mV increments. To subtract the leak current, the hyperpolarizing prepulse was omitted and instead the prepulse was set to −40 mV to remove *I*_A_ activation from the −50 mV holding potential. Currents were converted to conductance (*G* = *I*_peak_/(*V*_m_–*V*_rev_) and fitted to a first order Boltzmann equation. *V*_rev_ *I*_A_ = −86 mV (Eisen and Marder, 1982). TEVC experiments were done at 19–22^°^C as measured with a probe in the bath. Temperature did not change by more than 1^°^C during any given experiment.

### Cloning and sequencing lobster argonaute 1 (AGO1)

Total RNA was isolated from the lobster nervous system using TRIzol (Ambion, Austin, TX) and converted to cDNA using Superscript (Life Technologies, Grand Island, NY) according to manufacturers’ instructions. Degenerate primers were generated based on alignments with *Drosophila melanogaster* (Genbank accession: AB035447), *Penaeus monodon* (Genbank accession: DQ343133), and *Daphnia pulex* (wfleabase: NCBI_GNO_68324)** and are shown in Table 1. Degenerate polymerase chain reactions (PCRs) were performed with Advantage Taq (Clontech, Mountain View, CA) as previously described (Baro et al., 1994). PCR products were cloned with a TA cloning kit (Qiagen, Valencia, CA) using the manufacturer’s instructions. The 3′ end was obtained with lobster specific primers, S. For 1 (Table 1) and a SMARTer RACE kit (Clontech) using instructions provided. The 5′ end was obtained with lobster specific primer, S. Rev 2 (Table 1) and a FirstChoice RLM RACE Kit (Ambion) using instructions provided. All sequencing was performed by the GSU DNA core facility. Sequences were analyzed and manipulated with the Lasergene 10 suite of DNASTAR software (Madison, WI).

**Table 1:** **PCR Primers**.

Primer Description	Sequence 5’ to 3’
D. For 1	TKCARACDTCKRCYATGATCAA
D. Rev 1	TGHGTYACATCRGCWCCCA
D. For 2	CCIGAYAARTGYCCIMGIMRRGTNAA
S. For 1	GTCCCAGGCATCAGACCGAAGGTGTTC
S. Rev 1	CGAACCAAATTGTTTATCTCTCTCTCTCGGTCAGG
S. Rev 2	CTGGGAAAGGCATGTACCATGGTCTCG

### Peptide injection

The his-tagged hook (HHHHHHPDNGTSAWGEPNESSPGWGEMD) and mutant hook (HHHHHHPDNGTSvavEPNESSPvavEMD) peptides were diluted in water to a working concentration of 10 ng/ml and fast green was added to 0.04% to visualize injections. Microloaders (Eppendorf) were used to directly fill glass pipettes (8–15 MΩ when filled with 3 M KCl) with the solution (i.e., no backfilling). Because of the high resistance of the peptide solution, pipette tips were broken before injection by gently touching a Kim wipe. The peptide was pressure injected into LP neurons using a Picospritzer III (General Valve/Parker Hannifin). Only two pressure pulses (on average 32 psi and 47 ms) separated by 30 s were applied. Intracellular recording during the injection showed that the injection procedure had no effect on LP voltage envelope and firing properties. Extracellular recordings were used to continuously monitor the activity of the LP neuron before, during and for 1 h after peptide injection.

### Statistical analyses

The data were checked for normality and analyzed using parametric statistical tests including Student *t*-tests and ANOVAs. In the one case where data were not normally distributed, a non-parametric Kruskal-Wallis test was used. All data were analyzed using Prism Statistical software package (Graphpad). Significance threshold was set at *p* < 0.05 in all cases. Statistical outliers were excluded if the values fell greater than two standard deviations from the mean and this resulted in exclusion of one experiment. Means and standard errors are presented unless otherwise noted. ANOVAs were usually followed by Tukey’s *post hoc* tests that make all pairwise comparisons.

## Results

### Experimental model

**Figure 1:**
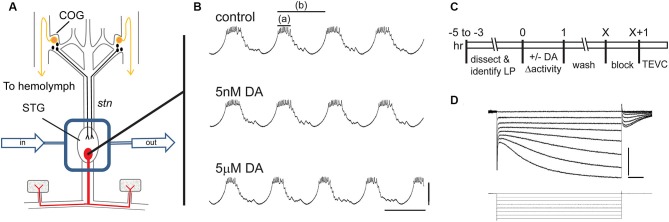
**The experimental model. (A)** The stomatogastric nervous system is dissected and pinned in a dish. Dopamine neurons (black) in the commissural ganglia (COGs) project through the stomatogastric nerve (*stn*) to the STG. The L-cells (gold) in the COGs are the source of neurohormonal DA that constantly bathes the STG. In these experiments, the STG is isolated with a Vaseline well (rectangle) and constantly superfused throughout the experiment (arrows). There are ~30 neurons in the STG including the single LP neuron that is illustrated in red. **(B)** Intracellular LP recordings from a typical experiment where the STG was sequentially superfused with saline (control), 5 nM DA and 5 µM DA. Note that 5 µM but not 5 nM produced a significant decrease in LP burst duration (a) and cycle period (b). Scale bars are 20 mV and 500 ms. **(C)** Diagram of typical somatic TEVC experiments to measure persistent changes in LP *I*_h_. **(D)** Representative LP *I*_h_ recording elicited with a series of hyperpolarizations from −50 mV to −120 mV in 10 mV increments from a holding potential of −50 mV; current (top) and voltage (bottom) traces are shown; scale bars are 5 nA and 500 ms.

A persistent activity-dependent increase in LP *I*_h_ *G*_max_ was elicited by two coincident events: an activation of high affinity LP D1Rs and a reduction in LP burst duration (Rodgers et al., 2011a). We used a simple experimental model to coincidently elicit these events and study the cellular processes involved in long-term intrinsic plasticity: the spiny lobster STNS was dissected and pinned in a dish (Figure 1A). The STG, which contains the LP neuron, was continuously superfused with saline. Intracellular and extracellular recordings were used to identify the sole LP neuron as described in Section Materials and Methods. Both *in vivo* (Heinzel et al., 1993) and *in situ* (Figure 1B), the LP neuron undergoes spontaneous slow oscillations in membrane potential (~20 mV at 1–2 Hz) with a burst of spikes riding on the depolarized plateau of each oscillation. The standard experimental protocol used to elicit the persistent increase in LP *I*_h_ *G*_max_ is diagrammed in Figure 1C. LP activity was altered during a 1 h application of DA followed by washout of DA. At the end of the wash, the preparation was superfused with blocking saline for 1 h to prevent spontaneous activity, and LP *I*_h_ was then measured with somatic TEVC (Figure 1D). We previously demonstrated that in the absence of DA, LP *I*_h_ *G*_max_ does not exhibit rapid activity-dependent changes (Krenz et al., 2013); and, measures of LP *I*_h_ before and after the block indicate that it does not change appreciably during the block (LP *I*_h_ *G*_max_ before block = 0.125 + 0.013 µS; LP *I*_h_ *G*_max_ after 1 h block = 0.120 + 0.012 µS, *n* = 7, Student *t*-test, *p* = 0.796).

Three methods were previously used to elicit a persistent ~55% increase in LP *I*_h_ *G*_max_ by simultaneously activating high affinity D1Rs while altering LP activity (Rodgers et al., 2011a). The first two methods used a 1 h application of 5 nM DA to activate high affinity D1Rs and either concurrent application of TTX to block activity or concurrent injection of a hyperpolarizing bias current into LP to reduce LP burst duration and decrease LP duty cycle (burst duration/period). These treatments were followed by a 2.5 h saline wash, a 1 h block and TEVC measurement of LP *I*_h_. The fact that both methods produced the same persistent ~55% increase in LP *I*_h_ *G*_max_ suggested that the specific change in activity did not determine the magnitude of the alteration in LP *I*_h_ *G*_max_ measured 3.5 h after the treatment ended (other time points were not examined). In the absence of a change in activity, 5 nM DA had no effect; and, TTX had no significant effect in the absence of 5 nM DA. The third method used to elicit a persistent ~55% increase in LP *I*_h_ *G*_max_ was a 1 h application of 5 µM DA alone, which activates both high affinity D1Rs to permit activity-dependent regulation of LP *I*_h_ *G*_max_ and low affinity D1Rs to decrease LP burst duration and reduce LP duty cycle (Figure 1B, compare top and bottom panels). When TTX was included with 5 µM DA, the same 55% increase in LP *I*_h_ *G*_max_ was observed, again suggesting that the magnitude of the persistent change in LP *I*_h_ *G*_max_ measured 3.5 h after the treatment was not strictly correlated with the magnitude of the change in activity. However, a change in activity was required because, if a depolarizing bias current was injected into LP to prevent the 5 µM DA-induced decrease in LP burst duration and duty cycle, then there was no persistent change in LP *I*_h_ *G*_max_ in the presence of 5 µM DA. The most parsimonious interpretation of these data is that 5 µM DA and 5 nM DA + TTX acted through the same pathway to produce a persistent ~55% increase in LP *I*_h_ *G*_max_. Therefore, these two treatments are used interchangeably to study the processes involved.

### Time course of the persistent increase in lateral pyloric (LP) *I*_h_ *G*_max_

**Figure 2:**
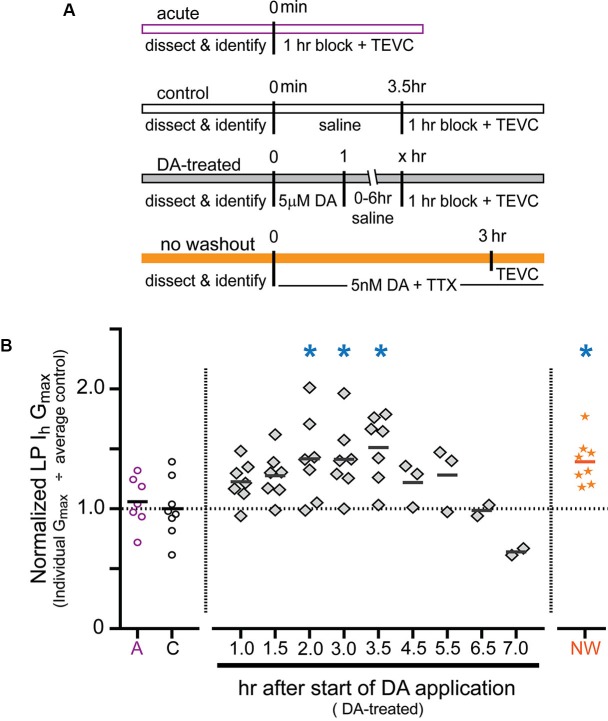
**Time course for the persistent increase in LP *I*_h_*G*_max_. (A)** Diagram of the experimental protocol for each of the four treatment groups. For all treatment groups a single measure was obtained for each preparation using two-electrode voltage clamp (TEVC) at the end of the experiment, i.e., LP *I*_h_ was not repeatedly measured over time within a given preparation; rather, terminal measurements from DA-treated preparations were compared to terminal measurements from control preparations and 68 animals were used for all of the experiments shown. Note that for the DA-treated group, the length of the saline wash varied across time points. **(B)** Plot of normalized LP *I*_h_ *G*_max_ for each experiment in every treatment group. Each symbol is a discrete experiment; e.g., the preparations in the 1 h DA-treatment group are different from the preparations in the 1.5 h DA-treatment group. Each *y*-value represents the LP *I*_h_ *G*_max_ for that experiment divided by the mean for the control experiments. The solid horizontal lines represent the means. Note that means will not be accurate at later time points where *n* ≤ 3, and they are only meant to show a decreasing trend over time. The numbers on the *x*-axis correspond to the hours that elapsed between the beginning of the DA application and the beginning of the block, i.e., *x* = 1 means that there was no saline wash before application of blocking saline; *x* = 2 indicates a 1 h saline wash, etc. Blue asterisks indicate significant differences relative to the control group as determined with a one-way ANOVA followed by Dunnett’s *post hoc* tests that compared the control treatment group to the acute and no washout treatment groups and each time point in the DA-treated group except those time points with *n* ≤ 3: *F*_(7,50)_ = 3.921, *p* = 0.0018.

Previous experiments showed that a 1 h DA application accompanied by a change in activity produced a 55% increase in LP *I*_h_ *G*_max_ measured after a 2.5 h DA washout followed by a 1 h block (Rodgers et al., 2011a). To gain insight into the mechanism involved, we examined the time course of the increase. The experiments are diagrammed in Figure 2A. For the DA-treated group, the STG was superfused with 5 µM DA for 1 h followed by washout with saline for 0–6 h. At the end of the washout, the STG was treated with blocking saline for 1 h followed by TEVC to measure LP *I*_h_. Control experiments were performed in which the STG was superfused with saline for 0 h (acute) or 3.5 h (control) followed by a 1 h block and TEVC to measure LP *I*_h_. The measured LP *I*_h_ *G*_max_ for each experiment was divided by the mean LP *I*_h_ *G*_max_ value for control experiments, and the resulting normalized LP *I*_h_ *G*_max_ was plotted (Figure 2B). The data indicated that the increase in LP *I*_h_ *G*_max_ developed slowly, peaked within 2–3 h of the start of DA application and then slowly declined over a similar time course. In the absence of 5 µM DA, LP *I*_h_ *G*_max_ did not change significantly over time (compare acute and control treatment groups).

In order to further demonstrate that the persistent activity-dependent increase in LP *I*_h_ was enabled by activation of high affinity D1Rs, and not washout of 5 µM DA, we performed one additional experiment (Figure 2A, orange bar). After dissection and cell identification, STGs were superfused with 5 nM DA + TTX for 3 h followed immediately by TEVC measures of LP *I*_h_. The data were normalized as described above and plotted (Figure 2B, orange stars). The results indicated that the persistent increase in LP *I*_h_ *G*_max_ did not depend upon DA washout. The mean fold-changes in LP *I*_h_ *G*_max_ for the two 3 h treatment groups were 1.39 + 0.07 (3 h 5 nM DA + TTX) vs. 1.42 + 0.14 (1 h 5 µM DA + 1 h wash + 1 h block). These means were not significantly different from one another, but both were significantly increased relative to control. Since we previously showed that neither 5 nM DA nor a change in LP activity produced a significant long-term change in LP *I*_h_ *G*_max_ relative to saline controls on its own (Rodgers et al., 2011a), we interpret the data presented here to mean that tonic activation of high affinity D1Rs enables a slow cellular process(es) that permits activity-dependent regulation of LP *I*_h_ G_max_.

### The type 1 DA receptor (D1R)- protein kinase a (PKA) axis is required for the persistent increase in lateral pyloric (LP) *I*_h_ *G*_max_

**Figure 3:**
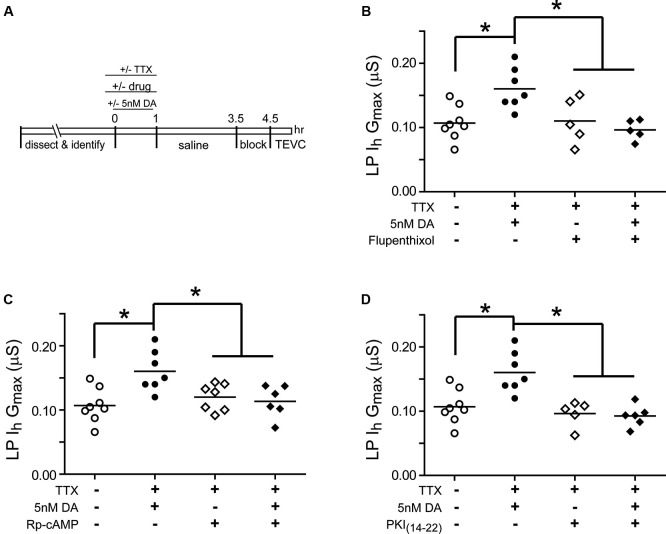
**A functional D1R-PKA axis is necessary to permit the persistent activity-dependent increase in LP *I*_h_ G_max_. (A)** Diagram of the experimental protocol. **(B)** The D1R inhibitor, flupenthixol (10 µM), had no effect on its own, but prevented the DA- and activity-dependent persistent increase in LP *I*_h_ *G*_max_. LP *I*_h_ *G*_max_ is plotted for every treatment group; each symbol represents one experiment, and the horizontal bars represent the means. Asterisks indicate significant differences as determined using a one-way ANOVA with Tukey’s *post hoc* tests that made all pairwise comparisons: *F*_(3,21)_ = 6.642, *p* = 0.0025. **(C)** The PKA inhibitor, Rp-cAMPS (1 mM), had no effect on its own, but prevented the DA- and activity-dependent persistent increase in LP *I*_h_ *G*_max_. Asterisks indicate significant differences as determined using a one-way ANOVA with Tukey’s multiple comparisons *post hoc* tests: *F*_(3,24)_ = 5.9, *p* = 0.0036. **(D)** The PKA inhibitor, myristoylated PKI_(14–22)_ (5 µM), prevented the DA- and activity-dependent persistent increase in LP *I*_h_ *G*_max_, but had no effect on its own. Asterisks indicate significant differences as determined using a one-way ANOVA with Tukey’s multiple comparisons *post hoc* tests: *F*_(3,22)_ = 10.38, *p* = 0.0002.

Experiments were next performed to determine if the persistent increase in LP *I*_h_ *G*_max_ was mediated by high affinity D1Rs acting through PKA (Figure 3). The experiment is diagrammed in Figure 3A: from *t* = −10–60 min, the STG was superfused with saline that in some cases contained TTX with or without a pharmacological reagent. In some experiments, 5 nM DA was added to the superfusate from *t* = 0–60 min. From *t* = 1 h–3.5 h, the STG was superfused with saline alone. The preparation was then blocked for 1 h and LP *I*_h_ was measured with TEVC. Previous work showed that under these conditions, superfusing TTX alone from *t* = −10–60 min had no significant effect on LP *I*_h_ *G*_max_ relative to saline controls (Rodgers et al., 2011a). Flupenthixol antagonizes LP D1Rs (Zhang et al., 2010; Rodgers et al., 2011b) and in these experiments10 µM flupenthixol blocked the increase in LP *I*_h_ *G*_max_ elicited by 5 nM DA + TTX but had no effect on its own (Figure 3B). Similarly, a competitive antagonist for cAMP binding to PKA, Rp-cAMPS, completely blocked the DA- and activity-dependent persistent increase in LP *I*_h_ *G*_max_, but had no effect in the absence of DA (Figure 3C). These data are consistent with the idea that D1Rs act through PKA to persistently alter LP *I*_h_ *G*_max_; however, Rp-cAMPS can potentially antagonize other cAMP binding proteins including exchange protein activated by cAMP (epac) and hyperpolarization activated cyclic nucleotide-gated (HCN) channels (Shabb, 2011). To confirm PKA involvement, the experiment was repeated with the specific membrane permeable PKA blocker, myristoylated PKI_(14–22)_, which specifically binds to and inactivates the catalytic subunit of PKA (Wen and Taylor, 1994; Shabb, 2011). PKI also blocked the DA- and activity-dependent persistent increase in LP *I*_h_ *G*_max_ but had no effect in the absence of DA (Figure 3D). Together these data suggested that a functional D1R-PKA axis was necessary for the persistent activity-dependent increase in LP *I*_h_ *G*_max_.

### Mechanistic target of rapamycin (mTOR)-dependent translation is required for the persistent increase in lateral pyloric (LP) *I*_h_ *G*_max_

**Figure 4:**
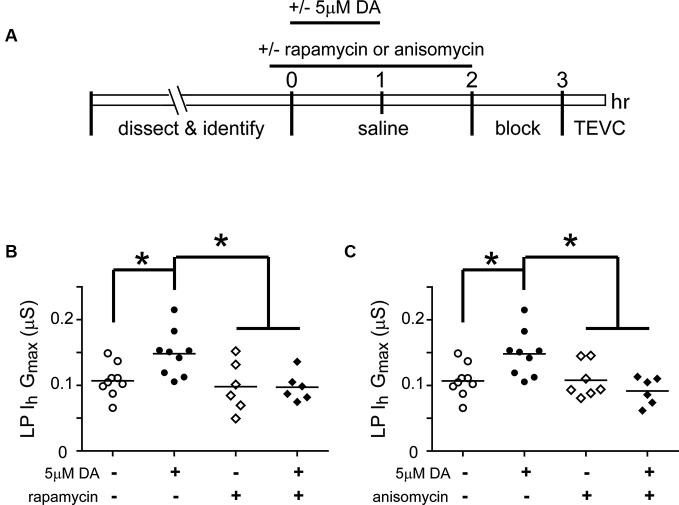
**The DA- and activity-dependent persistent increase in LP *I*_h_*G*_max_ is mediated by an mTOR-dependent translational mechanism. (A)** Diagram of the experimental protocol. **(B)** The mTORC1 inhibitor, rapamycin (100 nM), prevented the increase in LP *I*_h_ *G*_max_ normally elicited by 5 µM DA but had no effect on its own. LP *I*_h_ *G*_max_ is plotted for each treatment group; each symbol represents one experiment; the horizontal bars represent the means. Asterisks indicate significant differences as determined using a one-way ANOVA with Tukey’s *post hoc* tests that made all pairwise comparisons, *F*_(3,26)_ = 5.015, *p* = 0.0071.** (C)** The translation inhibitor, anisomycin (30 µM), had no effect on its own but prevented the persistent increase in LP *I*_h_ *G*_max_ elicited by 5 µM DA. Asterisks indicate significant differences as determined using a one-way ANOVA with Tukey’s multiple comparison *post hoc* tests, *F*_(3,27)_ = 5.976, *p* = 0.0029.

Mechanistic target of rapamycin (mTOR) is a conserved serine threonine kinase that functions as part of the protein complex, mTORC1, to regulate cap-dependent translation in all eukaryotic cells (Foster and Fingar, 2010). We used the mTORC1 specific blocker, rapamycin, and the translation blocker, anisomycin, to determine if mTORC1 and translation were also necessary for the DA- and activity-dependent increase in LP *I*_h_ *G*_max_ (Figure 4). In these experiments, from *t* = 0–60 min, the STG was superfused with saline that did or did not (control) contain 5 µM DA, followed by a 1 h wash with saline, a 1 h block and TEVC to measure LP *I*_h_. Either 100 nM rapamycin or 30 µM anisomycin was also superfused from *t* = −10–120 min. The data indicated that both mTOR and translation were necessary to produce the DA- and activity-dependent persistent increase in LP *I*_h_ *G*_max_. In the presence of either blocker, 5 µM DA could no longer elicit a significant increase in LP *I*_h_ *G*_max_, but the blockers had no effect on their own.

### The RNAi pathway is required for the persistent increase in lateral pyloric (LP) *I*_h_ *G*_max_

**Figure 5:**
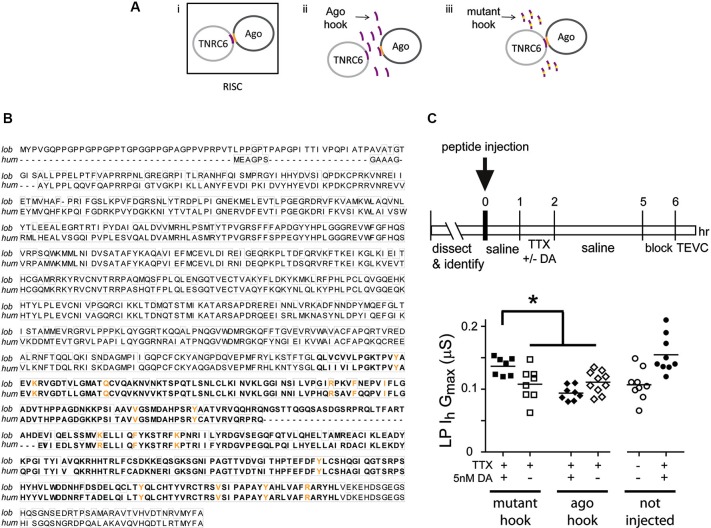
**A functional RNAi pathway is necessary for the DA- and activity-dependent increase in LP *I*_h_*G*_max_. (A)** *i.* The Ago hook peptide on the TNRC6 protein (purple) binds amino acids in the PIWI domain of the Ago protein (orange); the dimer is a component of the multiprotein complex, RISC, which is an essential element in the RNAi pathway. Additional RISC proteins are not shown. *ii.* Ago hook peptide competes with TNRC6 for binding to Ago, and excess Ago hook peptide disrupts RISC formation and the RNAi pathway. *iii.* Mutating amino acids in the Ago hook prevents it from binding to Ago, and the TNRC6-Ago dimer forms in the presence of excess mutant hook. **(B)** Alignment of lobster (KF602070) and human (AF093097) Ago1 proteins. Identical amino acids are boxed. The PIWI domain involved in binding TNRC6 is bolded. Amino acids necessary for binding to TNRC6 are shown in orange. **(C)** Injecting hook, but not mutant hook peptide into the LP neuron prevented the DA-induced, activity-dependent persistent increase in LP *I*_h_ *G*_max_. The upper panel shows the experimental protocol. The lower panel plots LP *I*_h_ *G*_max_ for each treatment. Each symbol is one experiment; the horizontal bars are the means. Asterisk indicates significant differences using a one-way ANOVA with Tukey’s *post hoc* tests that made all pairwise comparisons, *F*_(3,29)_ = 7.036, *p* = 0.0011. Uninjected control and DA-treated preparations from experiments in Figure 3 are shown for comparison.

Activity-dependent intrinsic plasticity involving mTORC1 often requires additional regulatory elements that bind mRNA, including microRNA(s) (miRs) (Goldie and Cairns, 2012). The RNAi pathway processes miRs and mediates their actions (Finnegan and Pasquinelli, 2013). We next asked if a functional RNAi pathway was necessary for the persistent DA- and activity-dependent increase in LP *I*_h_ *G*_max_. The experimental logic is diagrammed in Figure 5A. The RNA interference silencing complex (RISC) is an essential component of the RNAi pathway. RISC comprises several proteins including members of the Argonaute (Ago) and TNRC6/GW182 families. Dimerization occurs between members of the Ago and TNRC6 families, and disrupting this interaction prevents RISC formation and blocks the RNAi pathway and miR effects (Till et al., 2007). The minimal Ago binding domain from TNRC6 proteins has been identified as a continuous stretch of 22 amino acids, termed the Ago hook (Figure 5A, purple). An excess of the hook peptide can outcompete endogenous TNRC6 proteins for binding to endogenous Ago1 and 2 in human tissue culture cell lines (Till et al., 2007) (Figure 5A, panel ii). Altering amino acids in the Ago hook (termed mutant hook) prevented it from binding to Ago. Ago and TNRC6 proteins dimerized in the presence of an excess of the mutant hook (Figure 5A, panel iii). Ago is highly conserved across species, and the human Ago hook has been used successfully to disrupt the effects of a *Drosophila* miR in an *in vitro* translation assay and to isolate *Drosophila* Ago1 and yeast Ago in pull-down assays (Till et al., 2007). The Ago amino acids that are necessary to bind the Ago hook have been identified (Till et al., 2007), and are indicated in orange in Figures 5A, B. In order to determine if the amino acids involved in binding the Ago hook were conserved in lobster, we cloned lobster Ago1, which shares 83% identity with *Drosophila* Ago1, and compared it to each of the four human Ago proteins. These comparisons indicated that lobster Ago1 was ≥72% identical to each human Ago. An alignment of lobster and human Ago1 proteins indicated that they shared 74% identity over their entire length; and, 16 of the 17 amino acids known to be involved in binding the Ago hook were identical with the single amino acid change being conservative (Figure 5B). Together, the existing data suggested that the previously validated human Ago hook and mutant hook peptides could be used in our experiments to test if a functional RNAi pathway was necessary for the persistent increase in LP *I*_h_ *G*_max_.

Experiments involving peptide injections into LP neurons are shown in Figure 5C. We pressure injected hook or mutant hook peptides into LP neurons as described in Section Materials and Methods. The STG was then superfused for 1 h to allow the injected peptide to compete with endogenous proteins for binding to LP Ago1. No change in rhythmic LP activity was observed during or after peptide injection. We next superfused the STG with 5 nM DA + TTX or TTX (control) for 1 h, followed by a 3 h wash with saline, a 1 h block and TEVC to measure LP *I*_h_. The data indicated that peptide injections had no effect on LP *I*_h_ *G*_max_. In the absence of 5 nM DA, LP *I*_h_ *G*_max_ was not significantly different between uninjected neurons and neurons injected with Ago hook or mutant hook peptides (one-way ANOVA, *F*_(2,23)_ = 0.3245, *p* = 0.7264). On the other hand, injection of the Ago hook, but not the mutant hook, prevented the usual persistent increase in LP *I*_h_ *G*_max_ in the presence of 5 nM DA + TTX (Figure 5C). These data indicated that the RNAi pathway was necessary to elicit the DA- and activity-dependent persistent increase in LP *I*_h_ *G*_max_.

### Transcription is necessary for the Dopamine (DA)- and activity-dependent persistent increase in lateral pyloric (LP) *I*_h_ *G*_max_

**Figure 6:**
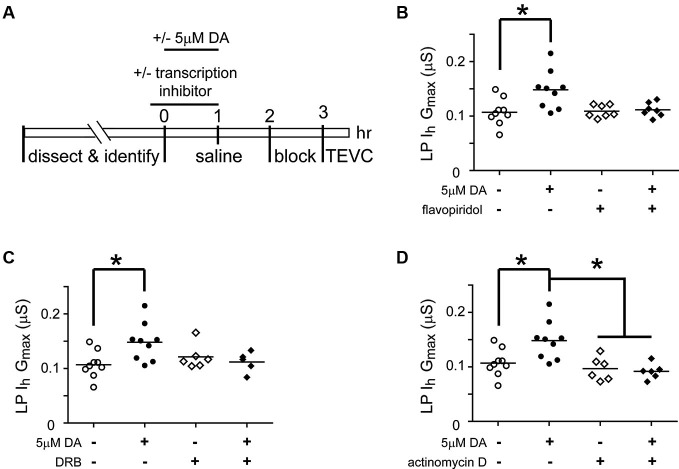
**Transcription is required for the DA- and activity-dependent persistent increase in LP *I*_h_*G*_max_. (A)** Diagram of the experimental protocol. **(B)** Flavopiridol (100 nM) blocks the persistent increase in LP *I*_h_ *G*_max_ elicited by 5 µM DA. LP *I*_h_ *G*_max_ is plotted for each treatment group; each symbol is one experiment; the horizontal bars represent the means. Asterisk indicates a significant difference as determined using *t*-tests to compare DA and saline treatment groups in preparations with (*p* = 0.701) and without (*p* = 0.011) flavopiridol. Note that an ANOVA could not be performed due to unequal variances between +/− flavopiridol groups (*F*-test, *p* < 0.03). **(C)** DRB (100 µM) blocks the persistent increase in LP *I*_h_ *G*_max_ elicited by 5 µM DA. Asterisk indicates a significant difference as determined using a one-way ANOVA with Tukey’s *post hoc* tests that made all pairwise comparisons, *F*_(3, 25)_ = 3.827, *p* < 0.022. **(D)** Actinomycin D (50 µM) blocks the persistent increase in LP *I*_h_ *G*_max_ elicited by 5 µM DA but has no effect alone. Asterisks indicate significant differences as determined using a one-way ANOVA with Tukey’s *post hoc* tests that made all pairwise comparisons, F_(3, 26)_ = 7.611, *p* = 0.0008.

Activity-dependent processes can involve transcriptional regulation of mRNAs and/or miRs (Krol et al., 2010; Wibrand et al., 2010; Kandel, 2012). RNA polymerase II transcribes both mRNAs and miRs (Pawlicki and Steitz, 2010). In order to determine if RNA Polymerase II-dependent transcription was necessary for the DA- and activity-dependent increase in LP *I*_h_ *G*_max_, we first employed pharmacological agents that acted on RNA Polymerase II to prevent transcription (Figure 6). The STG was superfused with or without (control) 5 µM DA from *t* = 0–60 min. This was followed by a 2 h wash with saline, then a 1 h block and TEVC to measure LP *I*_h_. Either 100 nM flavopiridol or 100 µM was superfused from *t* = −10–60 min. The results indicated that the drugs blocked the persistent increase in LP *I*_h_ *G*_max_ (Figures 6B, C). These drugs act by inhibiting cyclin dependent kinases (CDKs) that phosphorylate RNA polymerase II and thereby promote transcript elongation (Bensaude, 2011); however, CDKs are known to regulate a number of other proteins. For this reason, we repeated the experiments with a third transcription blocker, actinomycin D, which acts by intercalating into the DNA (Bensaude, 2011). Inclusion of 50 µM actinomycin D in the superfusate had no effect on its own, but blocked the DA- and activity-dependent persistent increase in LP *I*_h_ *G*_max_ (Figure 6D). Together these data suggested that RNA Polymerase II transcription was necessary for the DA- and activity-dependent increase in LP *I*_h_ *G*_max_. Finally, to test the previously stated assumption that the persistent effects of 5 nM DA + TTX and 5 µM DA on LP *I*_h_ *G*_max_ were mediated by the same pathway, we repeated the flavopiridol experiment with 5 nM DA + TTX. Consistent with our hypothesis, flavopiridol blocked the persistent 55% increase in LP *I*_h_ *G*_max_ elicited by 5 nM DA + TTX ( mean + SEM LP *I*_h_ *G*_max_ in 5 nM DA + TTX = 0.155 + 0.01 µS, *n* = 9; in flavopiridol + 5 nM + TTX, = 0.108 + 0.008 µS, *n* = 4; Student’s *t*-test *p* = 0.015)

### The same slow processes are necessary for the 5 nM DA induced, activity-independent increase in lateral pyloric (LP) *I*_h_ *G*_max_

**Figure 7:**
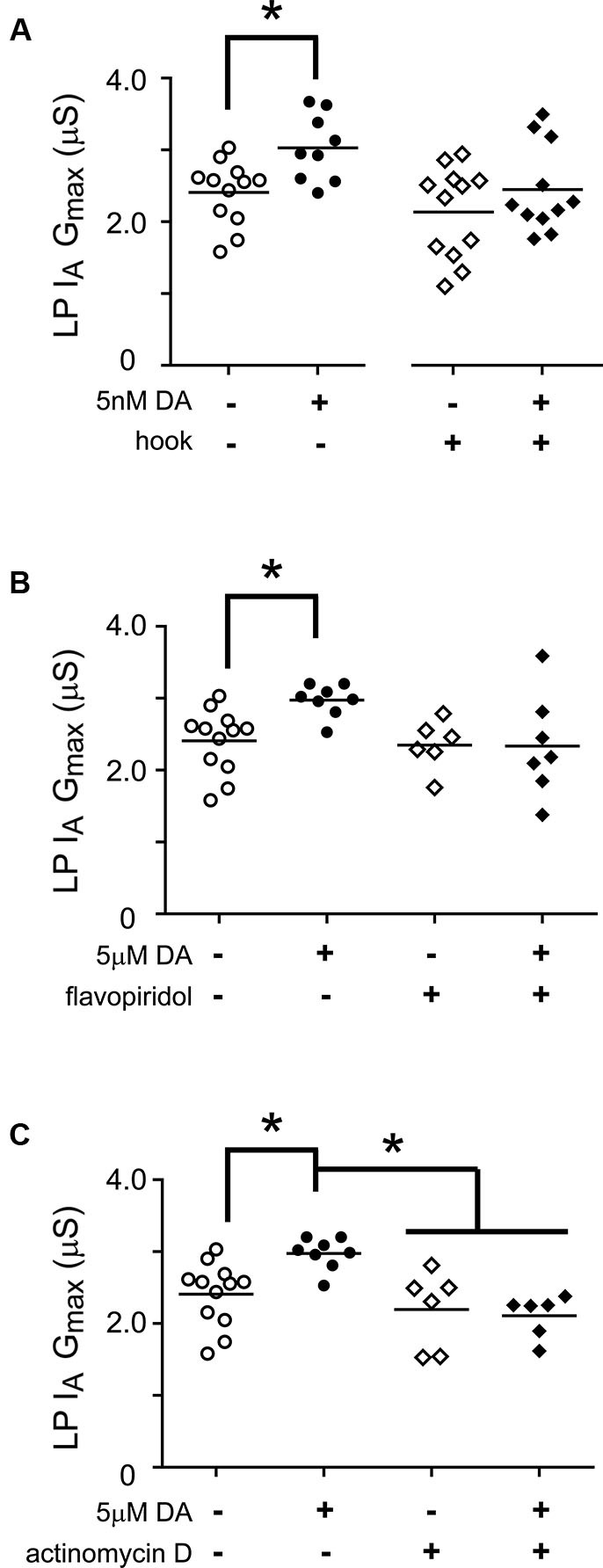
**miR transcription is required for the DA-dependent persistent increase in LP *I*_h_*G*_max_. (A)** The Ago hook blocks the persistent increase in LP* I*_A_ *G*_max_ elicited by 5 nM DA. The Ago hook injection experiments described in Figure 5A were repeated without TTX, and LP *I*_A_ *G*_max_ was measured. Each symbol represents one experiment, and the horizontal bars represent the means. The asterisk indicates a significant difference between the saline and DA-treated preparations, as determined using Student *t*-tests for the non-injected preparations (*p* < 0.006) and the hook-injected preparations (*p* = 0.244). **(B)** Flavopiridol (100 nM) blocked the persistent increase in LP *I*_A_ *G*_max_ elicited by 5 µM DA. Experiments diagrammed in Figure 6A were repeated with flavopiridol except that LP *I*_A_ was measured and plotted for each treatment group. Each symbol is one experiment; horizontal bars are the means. Asterisk indicates a significant difference as determined using *t*-tests to compare DA and saline treatment groups in preparations with (*p* = 0.969) and without (*p* = 0.004) flavopiridol. Note that unequal variances between +/− flavopiridol groups prevented analysis with an ANOVA (*F*-test, *p* < 0.008). **(C)** Actinomycin D (50 µM) blocked the persistent increase in LP *I*_A_ *G*_max_ elicited by 5 µM DA. Experiments diagrammed in Figure 6A were repeated with Actinomycin D, except that LP *I*_A_ was measured and plotted for each treatment group. Each symbol is one experiment; horizontal bars are the means. Asterisks indicate significant differences as determined using a Kruskal-Wallis test with Dunn’s multiple comparison posthoc tests, *p* = 0.0014.

Thus far we have examined the cellular processes underpinning the persistent increase in LP *I*_h_ *G*_max_ without regard for other voltage-gated ionic conductances; however, LP *I*_A_ and *I*_h_ can be co-regulated (MacLean et al., 2005; Temporal et al., 2012; Krenz et al., 2013). We previously demonstrated that a 1 h application of 5 nM DA produced a persistent increase in LP *I*_A_ that was dependent upon the D1R-PKA axis and mTOR-dependent translation; but, unlike LP *I*_h_, the persistent increase in LP *I*_A_ was activity-independent (Rodgers et al., 2011b, Rodgers et al., 2013). In order to better understand the signaling network that co-regulates LP *I*_A_ and *I*_h_, we asked if RNAi and transcription were also necessary for the activity-independent persistent increase in LP *I*_A_ G_max_ (Figure 7). We repeated the hook injection experiment diagrammed in Figure 5A using 5 nM DA without TTX and measured LP *I*_A_. The hook blocked the DA induced increase in LP *I*_A_ *G*_max_ (Figure 7A); thus, the RNAi pathway was necessary for the persistent increase in LP *I*_A_. We next repeated the experiments with the transcription blockers diagrammed in Figure 6A. DRB alone significantly increased LP *I*_A_ *G*_max_ relative to saline controls (*t*-test, *p* = 0.026, *n* > 5 per treatment group), and was not considered further. On the other hand, both flavopiridol (Figure 7B) and actinomycin D (Figure 7C) blocked the DA-induced increase in LP *I*_A_ *G*_max_. Consistent with the idea that 5 µM DA and 5 nM DA acted through the same pathway, flavopiridol also blocked the persistent ~25% increase in LP *I*_A_ *G*_max_ elicited by 5 nM DA + TTX (mean + SEM LP I_A_ G_max_ in 5 nM DA + TTX = 3.1 + 0.2 µS, *n* = 8; in flavopiridol + 5 nM + TTX = 2.08 + 0.23 µS, *n* = 4; Student’s *t*-test *p* = 0.005). We concluded that RNA polymerase II transcription was also necessary for the DA-induced persistent increase in LP *I*_A_.

## Discussion

The main finding of the work presented here is that tonic nM DA can act at high affinity D1Rs to permit a persistent, activity-dependent increase in LP *I*_h_ *G*_max_ through a signaling network that relies on the canonical D1R-PKA axis, RNA Polymerase II transcription, components of the RNAi pathway, mTORC1 and translation. All of these same elements are also necessary for the activity-independent, persistent increase in LP *I*_A_ *G*_max_ elicited by tonic nM DA.

### Potential mechanisms for how 5 nM DA persistently regulates lateral pyloric (LP) *I*_A_ and LP *I*_h_

Modulatory tone continuously influences ion current density: Washout of modulatory tone reduced LP *I*_A_ *G*_max_ and adding 5 nM DA back to the bath prevented the decrease and could even produce a persistent increase (Rodgers et al., 2013). The mechanism involved did not rely on alterations in the number of Kv4 transcripts (Rodgers et al., 2011b) that encode the pore-forming subunits of the channels mediating LP *I*_A_ (Baro et al., 1997, 2000). If bath application of 5 nM DA was accompanied by a significant change in LP slow wave activity, then a persistent increase in LP *I*_h_ was also observed (Rodgers et al., 2011a). In the simplest case, high affinity D1Rs regulate both LP *I*_A_ and *I*_h_ through the same mechanism, and activity-dependence is bestowed upon LP *I*_h_ through an additional process.

RNA polymerase II transcription is essential for the persistent increase in LP *I*_A_ and *I*_h_ elicited by 5 nM DA. Both mRNAs and miRs are transcribed by RNA polymerase II. Our data suggest miR expression is regulated by dopaminergic tone. The RNAi pathway, which processes miRs and mediates their effects, is necessary for the DA-induced persistent increases in LP *I*_A_ and *I*_h_ *G*_max_. Injecting the Ago hook to sequester endogenous Ago1, and thereby obstruct RNAi, did not appear to alter LP *I*_A_ or *I*_h_ over the long-term (several hours); however, Ago hook injections did block the persistent increase in LP *I*_A_ and *I*_h_ *G*_max_ elicited by 5 nM DA. The most parsimonious interpretation of these data is that DA regulates miR expression, although there are other explanations (Pinder and Smibert, 2013). If DA suppressed miR expression, then Ago hook injections should have occluded the DA effect. Since Ago hook injections blocked rather than occluded DA’s effect, it is more likely that DA enhanced miR expression. Consistent with this hypothesis, activation of high affinity D1Rs has been shown to enhance miR-181a expression in hippocampal neurons (Saba et al., 2012). The half-lives of miRs are variable, ranging from minutes to hours (Bail et al., 2010; Krol et al., 2010). MiR expression can be regulated by altering rates of transcription (Fiore et al., 2009; Impey et al., 2010; Nudelman et al., 2010), processing (Heinrich et al., 2013; Massirer and Pasquinelli, 2013) and/or degradation (Chatterjee and Grosshans, 2009; Krol et al., 2010; Wibrand et al., 2010; Grosshans and Chatterjee, 2011). DA could be acting on one or all three of these processes to enhance miR expression. The D1R-PKA axis could directly increase transcription rates through the cAMP response element binding protein (CREB), a transcription factor known to augment the expression of several miRs (Vo et al., 2005; Tan et al., 2012a,b). Monoamines can also regulate the expression of Piwi-interacting RNAs (piRs), an additional class of small noncoding RNAs that can promote long-term neuronal plasticity by regulating transcription factor expression (Rajasethupathy et al., 2012). Thus, it is possible that DA could indirectly influence miR transcription by regulating piRs. It should be noted that although both Piwi and Ago1 proteins possess PIWI domains, the amino acids necessary for binding to the Ago hook are not preserved in Piwi proteins (Parker et al., 2004), and the Ago hook does not pull down Piwi proteins (Till et al., 2007). Theoretically, DA could also regulate the processing or stabilization of nascent miRs, but to the best of our knowledge, this has not yet been demonstrated. For the remainder of this discussion, we assume the same miR(s) controls both LP *I*_A_ and *I*_h_ densities in order to permit their co-regulation; however, it is also possible that distinct miRs regulate LP *I*_A_ and *I*_h_ densities, and in this case, both DA and a change in activity may be required to increase the expresson of the miR regulating *I*_h_ density (Wibrand et al., 2010; Cohen et al., 2011; Eacker et al., 2011).

Both mTORC1 and translation are necessary for the DA-induced persistent increases in LP *I*_A_ and *I*_h_ *G*_max_. Many cellular processes are regulated by mTORC1, including cap-dependent translation (Laplante and Sabatini, 2012). The most parsimonious interpretation of the data is that DA directly or indirectly enhances mTORC1-dependent translation of a protein(s) because, the mTORC1 inhibitor, rapamycin, and the translation inhibitor, anisomycin, had no effect on their own, but each prevented the persistent increases in LP *I*_A_ and *I*_h_ elicited by 5 nM DA. The identity of the transcript(s) undergoing enhanced mTORC1-dependent translation is unknown. Increased translation of ion channel subunits could augment ion channel surface expression and LP maximal conductances, including the pore-forming subunits that mediate *I*_A_ (Kv4) and *I*_h_ (HCN) or the auxiliary subunits that regulate channel conductance and trafficking (An et al., 2000; Zhang et al., 2003; Santoro et al., 2009; Lin et al., 2010; Santoro et al., 2011). Additional candidates for altered translation include a wide variety of proteins involved in ion channel translation, trafficking and surface expression. Despite the fact that there are many potential targets, for the ease of discussion, here we further consider only Kv4 and HCN transcripts.

How might the increased expression of a miR lead to increased mTORC1-dependent translation of Kv4 and HCN transcripts? RNA binding proteins (RBPs) act in a combinatorial fashion to repress or enhance translation of the transcript to which they bind (Darnell and Richter, 2012; Darnell, 2013). miRs remodel the RBP complexes bound to transcripts and thereby either inhibit or facilitate their translation (Lee and Vasudevan, 2013). We hypothesize that 5 nM DA promotes expression of a miR that can reconfigure the RBP complexes on Kv4 and HCN transcripts to facilitate their translation. There are a number of ways that this could occur: the miR could act as a decoy and compete with Kv4 and HCN transcripts for binding to a repressive RBP (Eiring et al., 2010); or, the miR could compete with a more repressive RBP for binding to Kv4 and HCN transcripts (Ma et al., 2010). Then again, the miR could noncompetitively bind Kv4 and HCN transcripts and recruit RBPs that promote translation (Vasudevan et al., 2007; Tsai et al., 2009). Alternatively, the miR could de-repress Kv4 and HCN transcripts by reducing the number of available repressive RBPs; for example, the miR could bind repressive RBP transcripts and block their translation initiation (Djuranovic et al., 2012; Meijer et al., 2013) and/or elongation (Graber et al., 2013a) and/or promote their degradation (Djuranovic et al., 2011; Fukaya and Tomari, 2012). Since a given transcript is regulated by multiple elements, the aforementioned models could account for both the activity-dependent and -independent regulation of LP *I*_h_ and *I*_A_, respectively, if we postulate activity-dependent remodeling of an additional RBP complex on HCN transcripts. Although these hypotheses have the advantage of being simple and straightforward, they are highly speculative. It is also possible that the miR(s) indirectly alters RBP complexes on Kv4 and HCN transcripts by regulating transcripts encoding other types of proteins. For example, Kv1 transcripts in hippocampal neurons compete with CAMKIIα and other transcripts for binding to a limited number of Hu/embryonic lethal, abnormal vision (ELAV) RBPs that promote translation; and, Kv1 transcripts bind these facilitatory RBPs and are translated only when competitor transcripts (e.g., CAMKIIα are destabilized and degraded (Sosanya et al., 2013), suggesting that the shared RBPs may promote switching between two distinct programs/states.

### Commonalities between activity-dependent regulation of lateral pyloric (LP) *I*_h_ and synaptic plasticity

Learning and memory depend upon coordinated intrinsic and synaptic plasticity (Sehgal et al., 2013). Coordination can be achieved through shared transduction components. In this regard, many of the cellular processes underpinning long-term activity-dependent regulation of LP *I*_h_ *G*_max_ and synaptic plasticity are similar. First, miRs can contribute to long-term synaptic plasticity in multiple species. Throughout the mammalian brain, miRs participate in activity-dependent synaptic remodeling and regulate cognition by controlling components of the post-synaptic density, spine volume and synaptic cytoskeletal proteins (Schratt, 2009; Eacker et al., 2013; Hansen et al., 2013). miRs are also linked to synaptic plasticity and long-term memory in *Drosophila* (Ashraf et al., 2006; McCann et al., 2011). In *Aplysia*, serotonin can down-regulate expression of a miR that normally constrains synaptic plasticity (Rajasethupathy et al., 2009). Second, mTOR-dependent translation is necessary for long-term synaptic plasticity in a number of systems (Hoeffer and Klann, 2009; Gkogkas et al., 2010; Graber et al., 2013b). In rat hippocampal neurons, the D1R-PKA axis permits local mTORC1-dependent translation of the glutamate receptor subunit, GluR1, in an activity-dependent fashion (Smith et al., 2005). D1Rs mediate memory consolidation in the gerbil auditory cortex through mTOR-dependent protein synthesis (Schicknick et al., 2008). In Aplysia, long-term facilitation of a sensory-motor synapse relies on serotonin-enabled local mTORC1-dependent translation (Yanow et al., 1998; Casadio et al., 1999; Wang et al., 2009). Similarly, long-term facilitation at a crayfish neuromuscular synapse required local mTOR-dependent translation (Beaumont et al., 2001). While synaptic and intrinsic activity-dependent processes employ similar mechanisms, it is important to note that modulatory tone also utilizes the same elements to persistently regulate ion current density in an activity-independent fashion (Rodgers et al., 2011b).

### Dopaminergic tone acts over two distinct time scales to co-regulate *I*_A_ and *I*_h_

The balance of ion conductances, rather than the absolute number of ion channels, can determine certain features of neuronal activity (Marder, 2011). It appears that several mechanisms can control the balance of the same conductance pair. Different mechanisms may predominate in each cell type; for example, GABA_A_ receptors and HCN1 channels co-vary to maintain hippocampal neuron resting membrane potential (Bonin et al., 2013), but in cortical pyramidal neurons, these two conductances vary inversely to maintain excitatory post synaptic potential summation (Chen et al., 2010). Even within the same cell type, two conductances can be co-regulated by multiple mechanisms that act over distinct time scales. In LP, *I*_A_ and *I*_h_ densities are coordinated by at least three distinct mechanisms in order to maintain the timing of LP activity; and, for two of the mechanisms, dopaminergic tone was shown to play a permissive role. In the first, most rapid mechanism, activation of high affinity D1Rs conferred activity-dependence upon LP *I*_h_. Alterations in LP activity that advanced LP firing phase largely due to a decrease in LP *I*_A_ triggered a rapid compensatory decrease in LP *I*_h_ to restore the timing of the LP activity phase (Krenz et al., 2013). Activation of high affinity LP D1Rs also enabled co-regulation of LP *I*_A_ and *I*_h_ through a second, slower process described here. Collectively, our work shows that an increase in dopaminergic tone produces a slow increase in LP *I*_A_ *G*_max_, independent of LP *I*_h_; however, when LP activity changes, then the same DA-enabled mechanism is engaged to increase LP *I*_h_ *G*_max_. In another study, overexpression of Kv4 channels in LP neurons increased LP *I*_A_ over days in organ culture and triggered a compensatory increase in LP *I*_h_ through a third, activity-independent mechanism (MacLean et al., 2003, 2005). Descending modulatory inputs were intact in the latter study, but it is unclear if modulatory tone played a role. It has been demonstrated that other modulators can maintain activity and conductance ratios over the long-term, and removal of modulators appears to change the ratios that are maintained (Rezer and Moulins, 1992; Thoby-Brisson and Simmers, 1998, 2002; Khorkova and Golowasch, 2007). Taken together, the data suggest that modulatory tone may influence neuronal identity by determining which homeostatic mechanisms are in play.

### Dopaminergic tone may persistently regulate voltage-gated conductances in other cell types

If regulation of voltage-gated conductances by modulatory tone is widespread, then the findings presented here could have important implications for neurological and psychiatric disorders involving disruptions in dopaminergic tone. For example, in a mouse model of Parkinson’s disease, dopaminergic tone was severely attenuated and *I*_h_ was persistently reduced in globus pallidus neurons (Chan et al., 2011). Since DA receptors are expressed in rodent globus pallidus neurons (Mansour et al., 1990; Marshall et al., 2001; Araki et al., 2007), the reduction in *I*_h_ could potentially be explained by a lack of normal DA-enabled, activity-dependent compensation.

## Conflict of interest statement

The authors declare that the research was conducted in the absence of any commercial or financial relationships that could be construed as a potential conflict of interest.

## References

[B1] An W. F. Bowlby M. R. Betty M. Cao J. Ling H. P. Mendoza G. (2000). Modulation of A-type potassium channels by a family of calcium sensors. Nature 403, 553–556 10.1038/35000592 10676964

[B2] Araki K. Y. Sims J. R. Bhide P. G. (2007). Dopamine receptor mRNA and protein expression in the mouse corpus striatum and cerebral cortex during pre- and postnatal development. Brain Res. 1156, 31–45 10.1016/j.brainres.2007.04.043 17509542 PMC1994791

[B3] Ashraf S. I. McLoon A. L. Sclarsic S. M. Kunes S. (2006). Synaptic protein synthesis associated with memory is regulated by the RISC pathway in Drosophila. Cell 124, 191–205 10.1016/j.cell.2005.12.017 16413491

[B4] Bail S. Swerdel M. Liu H. Jiao X. Goff L. A. Hart R. P. (2010). Differential regulation of microRNA stability. RNA 16, 1032–1039 10.1261/rna.1851510 20348442 PMC2856875

[B5] Baro D. J. Ayali A. French L. Scholz N. L. Labenia J. Lanning C. C. (2000). Molecular underpinnings of motor pattern generation: differential targeting of shal and shaker in the pyloric motor system. J. Neurosci. 20, 6619–6630 10964967 10.1523/JNEUROSCI.20-17-06619.2000 PMC6772986

[B6] Baro D. J. Cole C. L. Zarrin A. R. Hughes S. Harris-Warrick R. M. (1994). Shab gene expression in identified neurons of the pyloric network in the lobster stomatogastric ganglion. Receptors Channels 2, 193–205 7874446

[B7] Baro D. J. Levini R. M. Kim M. T. Willms A. R. Lanning C. C. Rodriguez H. E. (1997). Quantitative single-cell-reverse transcription-PCR demonstrates that A- current magnitude varies as a linear function of shal gene expression in identified stomatogastric neurons. J. Neurosci. 17, 6597–6610 9254672 10.1523/JNEUROSCI.17-17-06597.1997 PMC6573138

[B8] Beaumont V. Zhong N. Fletcher R. Froemke R. C. Zucker R. S. (2001). Phosphorylation and local presynaptic protein synthesis in calcium- and calcineurin-dependent induction of crayfish long-term facilitation. Neuron 32, 489–501 10.1016/s0896-6273(01)00483-4 11709159

[B9] Bensaude O. (2011). Inhibiting eukaryotic transcription: which compound to choose? How to evaluate its activity? Transcription 2, 103–108 10.4161/trns.2.3.16172 21922053 PMC3173647

[B10] Bonin R. P. Zurek A. A. Yu J. Bayliss D. A. Orser B. A. (2013). Hyperpolarization-activated current (In) is reduced in hippocampal neurons from Gabra5–/– mice. PLoS One 8:e58679 10.1371/journal.pone.0058679 23516534 PMC3597723

[B11] Casadio A. Martin K. C. Giustetto M. Zhu H. Chen M. Bartsch D. (1999). A transient, neuron-wide form of CREB-mediated long-term facilitation can be stabilized at specific synapses by local protein synthesis. Cell 99, 221–237 10.1016/s0092-8674(00)81653-0 10535740

[B12] Chan C. S. Glajch K. E. Gertler T. S. Guzman J. N. Mercer J. N. Lewis A. S. (2011). HCN channelopathy in external globus pallidus neurons in models of Parkinson’s disease. Nat. Neurosci. 14, 85–92 10.1038/nn.2692 21076425 PMC3058391

[B13] Chao S. H. Price D. H. (2001). Flavopiridol inactivates P-TEFb and blocks most RNA polymerase II transcription in vivo. J. Biol. Chem. 276, 31793–31799 10.1074/jbc.m102306200 11431468

[B14] Chatterjee S. Grosshans H. (2009). Active turnover modulates mature microRNA activity in Caenorhabditis elegans. Nature 461, 546–549 10.1038/nature08349 19734881

[B15] Chen X. Shu S. Schwartz L. C. Sun C. Kapur J. Bayliss D. A. (2010). Homeostatic regulation of synaptic excitability: tonic GABA(A) receptor currents replace I(h) in cortical pyramidal neurons of HCN1 knock-out mice. J. Neurosci. 30, 2611–2622 10.1523/JNEUROSCI.3771-09.2010 20164346 PMC2830721

[B16] Cleland T. A. Selverston A. I. (1995). Glutamate-gated inhibitory currents of central pattern generator neurons in the lobster stomatogastric ganglion. J. Neurosci. 15, 6631–6639 7472424 10.1523/JNEUROSCI.15-10-06631.1995 PMC6577986

[B17] Cohen J. E. Lee P. R. Chen S. Li W. Fields R. D. (2011). MicroRNA regulation of homeostatic synaptic plasticity. Proc. Natl. Acad. Sci. U S A 108, 11650–11655 10.1073/pnas.1017576108 21697510 PMC3136313

[B18] Darnell R. B. (2013). RNA protein interaction in neurons. Annu. Rev. Neurosci. 36, 243–270 10.1146/annurev-neuro-062912-114322 23701460 PMC3889695

[B19] Darnell J. C. Richter J. D. (2012). Cytoplasmic RNA-binding proteins and the control of complex brain function. Cold Spring Harb. Perspect. Biol. 4:a012344 10.1101/cshperspect.a012344 22723494 PMC3405866

[B20] Dixon D. Atwood H. L. (1989). Conjoint action of phosphatidylinositol and adenylate cyclase systems in serotonin-induced facilitation at the crayfish neuromuscular junction. J. Neurophysiol. 62, 1251–1259 2480994 10.1152/jn.1989.62.6.1251

[B21] Djuranovic S. Nahvi A. Green R. (2011). A parsimonious model for gene regulation by miRNAs. Science 331, 550–553 10.1126/science.1191138 21292970 PMC3955125

[B22] Djuranovic S. Nahvi A. Green R. (2012). miRNA-mediated gene silencing by translational repression followed by mRNA deadenylation and decay. Science 336, 237–240 10.1126/science.1215691 22499947 PMC3971879

[B23] Eacker S. M. Dawson T. M. Dawson V. L. (2013). The interplay of microRNA and neuronal activity in health and disease. Front. Cell. Neurosci. 7:136 10.3389/fncel.2013.00136 23986658 PMC3753455

[B24] Eacker S. M. Keuss M. J. Berezikov E. Dawson V. L. Dawson T. M. (2011). Neuronal activity regulates hippocampal miRNA expression. PLoS One 6:e25068 10.1371/journal.pone.0025068 21984899 PMC3184962

[B25] Eiring A. M. Harb J. G. Neviani P. Garton C. Oaks J. J. Spizzo R. (2010). miR-328 functions as an RNA decoy to modulate hnRNP E2 regulation of mRNA translation in leukemic blasts. Cell 140, 652–665 10.1016/j.cell.2010.01.007 20211135 PMC2924756

[B26] Erxleben C. F. deSantis A. Rathmayer W. (1995). Effects of proctolin on contractions, membrane resistance, and non-voltage-dependent sarcolemmal ion channels in crustacean muscle fibers. J. Neurosci. 15, 4356–4369 7540673 10.1523/JNEUROSCI.15-06-04356.1995 PMC6577732

[B27] Finnegan E. F. Pasquinelli A. E. (2013). MicroRNA biogenesis: regulating the regulators. Crit. Rev. Biochem. Mol. Biol. 48, 51–68 10.3109/10409238.2012.738643 23163351 PMC3557704

[B28] Fiore R. Khudayberdiev S. Christensen M. Siegel G. Flavell S. W. Kim T. K. (2009). Mef2-mediated transcription of the miR379-410 cluster regulates activity-dependent dendritogenesis by fine-tuning Pumilio2 protein levels. EMBO J. 28, 697–710 10.1038/emboj.2009.10 19197241 PMC2647767

[B29] Foster K. G. Fingar D. C. (2010). Mammalian target of rapamycin (mTOR): conducting the cellular signaling symphony. J. Biol. Chem. 285, 14071–14077 10.1074/jbc.R109.094003 20231296 PMC2863215

[B30] Fukaya T. Tomari Y. (2012). MicroRNAs mediate gene silencing via multiple different pathways in drosophila. Mol. Cell 48, 825–836 10.1016/j.molcel.2012.09.024 23123195

[B31] Gerfen C. R. Surmeier D. J. (2011). Modulation of striatal projection systems by dopamine. Annu. Rev. Neurosci. 34, 441–466 10.1146/annurev-neuro-061010-113641 21469956 PMC3487690

[B32] Gkogkas C. Sonenberg N. Costa-Mattioli M. (2010). Translational control mechanisms in long-lasting synaptic plasticity and memory. J. Biol. Chem. 285, 31913–31917 10.1074/jbc.R110.154476 20693284 PMC2952191

[B33] Goldie B. J. Cairns M. J. (2012). Post-transcriptional trafficking and regulation of neuronal gene expression. Mol. Neurobiol. 45, 99–108 10.1007/s12035-011-8222-0 22167484 PMC3259350

[B34] Graber T. E. Hebert-Seropian S. Khoutorsky A. David A. Yewdell J. W. Lacaille J. C. (2013a). Reactivation of stalled polyribosomes in synaptic plasticity. Proc. Natl. Acad. Sci. U S A 110, 16205–16210 10.1073/pnas.1307747110 24043809 PMC3791775

[B35] Graber T. E. McCamphill P. K. Sossin W. S. (2013b). A recollection of mTOR signaling in learning and memory. Learn. Mem. 20, 518–530 10.1101/lm.027664.112 24042848

[B36] Grosshans H. Chatterjee S. (2011). MicroRNases and the regulated degradation of mature animal miRNAs. Adv. Exp. Med. Biol. 700, 140–155 10.1007/978-1-4419-7823-3_12 21755479

[B37] Hansen K. F. Karelina K. Sakamoto K. Wayman G. A. Impey S. Obrietan K. (2013). miRNA-132: a dynamic regulator of cognitive capacity. Brain Struct. Funct. 218, 817–831 10.1007/s00429-012-0431-4 22706759 PMC3508255

[B38] Harris-Warrick R. M. Coniglio L. M. Levini R. M. Gueron S. Guckenheimer J. (1995). Dopamine modulation of two subthreshold currents produces phase shifts in activity of an identified motoneuron. J. Neurophysiol. 74, 1404–1420 8989381 10.1152/jn.1995.74.4.1404

[B39] Heinrich E. M. Wagner J. Kruger M. John D. Uchida S. Weigand J. E. (2013). Regulation of miR-17-92a cluster processing by the microRNA binding protein SND1. FEBS Lett. 587, 2405–2411 10.1016/j.febslet.2013.06.008 23770094

[B40] Heinzel H. G. Weimann J. M. Marder E. (1993). The behavioral repertoire of the gastric mill in the crab, Cancer pagurus: an in situ endoscopic and electrophysiological examination. J. Neurosci. 13, 1793–1803 8463850 10.1523/JNEUROSCI.13-04-01793.1993 PMC6576714

[B41] Hoeffer C. A. Klann E. (2009). mTOR signaling: at the crossroads of plasticity, memory and disease. Trends Neurosci. 33, 67–75 10.1016/j.tins.2009.11.003 19963289 PMC2821969

[B42] Howe M. W. Tierney P. L. Sandberg S. G. Phillips P. E. Graybiel A. M. (2013). Prolonged dopamine signalling in striatum signals proximity and value of distant rewards. Nature 500, 575–579 10.1038/nature12475 23913271 PMC3927840

[B43] Impey S. Davare M. Lasiek A. Fortin D. Ando H. Varlamova O. (2010). An activity-induced microRNA controls dendritic spine formation by regulating Rac1-PAK signaling. Mol. Cell. Neurosci. 43, 146–156 10.1016/j.mcn.2009.10.005 19850129 PMC2818337

[B44] Johnson B. R. Kloppenburg P. Harris-Warrick R. M. (2003). Dopamine modulation of calcium currents in pyloric neurons of the lobster stomatogastric ganglion. J. Neurophysiol. 90, 631–643 10.1152/jn.00037.2003 12904487

[B45] Kandel E. R. (2012). The molecular biology of memory: cAMP, PKA, CRE, CREB-1, CREB-2, and CPEB. Mol. Brain 5:14 10.1186/1756-6606-5-14 22583753 PMC3514210

[B46] Khorkova O. Golowasch J. (2007). Neuromodulators, not activity, control coordinated expression of ionic currents. J. Neurosci. 27, 8709–8718 10.1523/jneurosci.1274-07.2007 17687048 PMC3558984

[B47] Kiehn O. Harris-Warrick R. M. (1992). 5-HT modulation of hyperpolarization-activated inward current and calcium-dependent outward current in a crustacean motor neuron. J. Neurophysiol. 68, 496–508 1382120 10.1152/jn.1992.68.2.496

[B48] Kloppenburg P. Zipfel W. R. Webb W. W. Harris-Warrick R. M. (2007). Heterogeneous effects of dopamine on highly localized, voltage-induced Ca2+ accumulation in identified motoneurons. J. Neurophysiol. 98, 2910–2917 10.1152/jn.00660.2007 17728385

[B49] Krenz W. D. Hooper R. M. Parker A. R. Prinz A. A. Baro D. J. (2013). Activation of high and low affinity dopamine receptors generates a closed loop that maintains a conductance ratio and its activity correlate. Front. Neural Circuits 7:169 10.3389/fncir.2013.00169 24155696 PMC3805135

[B50] Krol J. Busskamp V. Markiewicz I. Stadler M. B. Ribi S. Richter J. (2010). Characterizing light-regulated retinal microRNAs reveals rapid turnover as a common property of neuronal microRNAs. Cell 141, 618–631 10.1016/j.cell.2010.03.039 20478254

[B51] Kuromi H. Kidokoro Y. (2000). Tetanic stimulation recruits vesicles from reserve pool via a cAMP-mediated process in Drosophila synapses. Neuron 27, 133–143 10.1016/s0896-6273(00)00015-5 10939337

[B52] Laplante M. Sabatini D. M. (2012). mTOR signaling in growth control and disease. Cell 149, 274–293 10.1016/j.cell.2012.03.017 22500797 PMC3331679

[B53] Lee S. Vasudevan S. (2013). Post-transcriptional stimulation of gene expression by microRNAs. Adv. Exp. Med. Biol. 768, 97–126 10.1007/978-1-4614-5107-5_7 23224967

[B54] Lin L. Sun W. Wikenheiser A. M. Kung F. Hoffman D. A. (2010). KChIP4a regulates Kv4.2 channel trafficking through PKA phosphorylation. Mol. Cell. Neurosci. 43, 315–325 10.1016/j.mcn.2009.12.005 20045463 PMC2823810

[B55] Ma F. Liu X. Li D. Wang P. Li N. Lu L. (2010). MicroRNA-466l upregulates IL-10 expression in TLR-triggered macrophages by antagonizing RNA-binding protein tristetraprolin-mediated IL-10 mRNA degradation. J. Immunol. 184, 6053–6059 10.4049/jimmunol.0902308 20410487

[B56] MacLean J. N. Zhang Y. Goeritz M. L. Casey R. Oliva R. Guckenheimer J. (2005). Activity-independent coregulation of IA and Ih in rhythmically active neurons. J. Neurophysiol. 94, 3601–3617 10.1152/jn.00281.2005 16049145

[B57] MacLean J. N. Zhang Y. Johnson B. R. Harris-Warrick R. M. (2003). Activity-independent homeostasis in rhythmically active neurons. Neuron 37, 109–120 10.1016/s0896-6273(02)01104-2 12526777

[B58] Mansour A. Meador-Woodruff J. H. Bunzow J. R. Civelli O. Akil H. Watson S. J. (1990). Localization of dopamine D2 receptor mRNA and D1 and D2 receptor binding in the rat brain and pituitary: an in situ hybridization-receptor autoradiographic analysis. J. Neurosci. 10, 2587–2600 2143777 10.1523/JNEUROSCI.10-08-02587.1990 PMC6570265

[B59] Marder E. (2011). Variability, compensation, and modulation in neurons and circuits. Proc. Natl. Acad. Sci. U S A 108, 15542–15548 10.1073/pnas.1010674108 21383190 PMC3176600

[B60] Marder E. Eisen J. S. (1984). Transmitter identification of pyloric neurons: electrically coupled neurons use different transmitters. J. Neurophysiol. 51, 1345–1361 6145757 10.1152/jn.1984.51.6.1345

[B61] Marshall J. F. Henry B. L. Billings L. M. Hoover B. R. (2001). The role of the globus pallidus D2 subfamily of dopamine receptors in pallidal immediate early gene expression. Neuroscience 105, 365–378 10.1016/s0306-4522(01)00180-4 11672604

[B62] Massirer K. B. Pasquinelli A. E. (2013). MicroRNAs that interfere with RNAi. Worm 2:e21835 10.4161/worm.21835 24058860 PMC3670461

[B63] McCann C. Holohan E. E. Das S. Dervan A. Larkin A. Lee J. A. (2011). The Ataxin-2 protein is required for microRNA function and synapse-specific long-term olfactory habituation. Proc. Natl. Acad. Sci. U S A 108, E655–E662 10.1073/pnas.1107198108 21795609 PMC3169144

[B64] Meijer H. A. Kong Y. W. Lu W. T. Wilczynska A. Spriggs R. V. Robinson S. W. (2013). Translational repression and eIF4A2 activity are critical for microRNA-mediated gene regulation. Science 340, 82–85 10.1126/science.1231197 23559250

[B65] Nirogi R. Komarneni P. Kandikere V. Boggavarapu R. Bhyrapuneni G. Benade V. (2013). A sensitive and selective quantification of catecholamine neurotransmitters in rat microdialysates by pre-column dansyl chloride derivatization using liquid chromatography-tandem mass spectrometry. J. Chromatogr. B Analyt. Technol. Biomed. Life Sci. 913–914, 41–47 10.1016/j.jchromb.2012.09.034 23270937

[B66] Nudelman A. S. DiRocco D. P. Lambert T. J. Garelick M. G. Le J. Nathanson N. M. (2010). Neuronal activity rapidly induces transcription of the CREB-regulated microRNA-132, in vivo. Hippocampus 20, 492–498 10.1002/hipo.20646 19557767 PMC2847008

[B67] Oginsky M. F. Rodgers E. W. Clark M. C. Simmons R. Krenz W. D. Baro D. J. (2010). D(2) receptors receive paracrine neurotransmission and are consistently targeted to a subset of synaptic structures in an identified neuron of the crustacean stomatogastric nervous system. J. Comp. Neurol. 518, 255–276 10.1002/cne.22225 19941347 PMC3956453

[B68] Owesson-White C. A. Roitman M. F. Sombers L. A. Belle A. M. Keithley R. B. Peele J. L. (2012). Sources contributing to the average extracellular concentration of dopamine in the nucleus accumbens. J. Neurochem. 121, 252–262 10.1111/j.1471-4159.2012.07677.x 22296263 PMC3323736

[B69] Parker J. S. Roe S. M. Barford D. (2004). Crystal structure of a PIWI protein suggests mechanisms for siRNA recognition and slicer activity. EMBO J. 23, 4727–4737 10.1038/sj.emboj.7600488 15565169 PMC535097

[B70] Park J. Takmakov P. Wightman R. M. (2011). In vivo comparison of norepinephrine and dopamine release in rat brain by simultaneous measurements with fast-scan cyclic voltammetry. J. Neurochem. 119, 932–944 10.1111/j.1471-4159.2011.07494.x 21933188 PMC3217157

[B71] Pawlicki J. M. Steitz J. A. (2010). Nuclear networking fashions pre-messenger RNA and primary microRNA transcripts for function. Trends Cell Biol. 20, 52–61 10.1016/j.tcb.2009.10.004 20004579 PMC2821161

[B72] Pinder B. D. Smibert C. A. (2013). microRNA-independent recruitment of Argonaute 1 to nanos mRNA through the Smaug RNA-binding protein. EMBO Rep. 14, 80–86 10.1038/embor.2012.192 23184089 PMC3537145

[B73] Rajasethupathy P. Antonov I. Sheridan R. Frey S. Sander C. Tuschl T. (2012). A role for neuronal piRNAs in the epigenetic control of memory-related synaptic plasticity. Cell 149, 693–707 10.1016/j.cell.2012.02.057 22541438 PMC3442366

[B74] Rajasethupathy P. Fiumara F. Sheridan R. Betel D. Puthanveettil S. V. Russo J. J. (2009). Characterization of small RNAs in Aplysia reveals a role for miR-124 in constraining synaptic plasticity through CREB. Neuron 63, 803–817 10.1016/j.neuron.2009.05.029 19778509 PMC2875683

[B75] Rezer E. Moulins M. (1992). Humoral induction of pyloric rhythmic output in lobster stomatogastric ganglion: in vivo and in vitro studies. J.Exp.Biol. 163, 209–230 1556513 10.1242/jeb.163.1.209

[B76] Rice M. E. Patel J. C. Cragg S. J. (2011). Dopamine release in the basal ganglia. Neuroscience 198, 112–137 10.1016/j.neuroscience.2011.08.066 21939738 PMC3357127

[B77] Rodgers E. W. Fu J. J. Krenz W. D. Baro D. J. (2011a). Tonic nanomolar dopamine enables an activity-dependent phase recovery mechanism that persistently alters the maximal conductance of the hyperpolarization-activated current in a rhythmically active neuron. J. Neurosci. 31, 16387–16397 10.1523/JNEUROSCI.3770-11.2011 22072689 PMC3758573

[B78] Rodgers E. W. Krenz W. D. Baro D. J. (2011b). Tonic dopamine induces persistent changes in the transient potassium current through translational regulation. J. Neurosci. 31, 13046–13056 10.1523/JNEUROSCI.2194-11.2011 21917788 PMC3544522

[B79] Rodgers E. W. Krenz W. D. Jiang X. Li L. Baro D. J. (2013). Dopaminergic tone regulates transient potassium current maximal conductance through a translational mechanism requiring D1Rs, cAMP/PKA, Erk and mTOR. BMC Neurosci. 14:143 10.1186/1471-2202-14-143 24225021 PMC3840709

[B80] Saba R. Storchel P. H. Aksoy-Aksel A. Kepura F. Lippi G. Plant T. D. (2012). Dopamine-regulated microRNA MiR-181a controls GluA2 surface expression in hippocampal neurons. Mol. Cell. Biol. 32, 619–632 10.1128/MCB.05896-11 22144581 PMC3266602

[B81] Santoro B. Hu L. Liu H. Saponaro A. Pian P. Piskorowski R. A. (2011). TRIP8b regulates HCN1 channel trafficking and gating through two distinct C-terminal interaction sites. J. Neurosci. 31, 4074–4086 10.1523/JNEUROSCI.5707-10.2011 21411649 PMC3077297

[B82] Santoro B. Piskorowski R. A. Pian P. Hu L. Liu H. Siegelbaum S. A. (2009). TRIP8b splice variants form a family of auxiliary subunits that regulate gating and trafficking of HCN channels in the brain. Neuron 62, 802–813 10.1016/j.neuron.2009.05.009 19555649 PMC2720631

[B83] Schicknick H. Schott B. H. Budinger E. Smalla K. H. Riedel A. Seidenbecher C. I. (2008). Dopaminergic modulation of auditory cortex-dependent memory consolidation through mTOR. Cereb. Cortex 18, 2646–2658 10.1093/cercor/bhn026 18321872 PMC2567422

[B84] Schratt G. (2009). microRNAs at the synapse. Nat. Rev. Neurosci. 10, 842–849 10.1038/nrn2763 19888283

[B85] Schultz W. (2007). Multiple dopamine functions at different time courses. Annu. Rev. Neurosci. 30, 259–288 10.1146/annurev.neuro.28.061604.135722 17600522

[B86] Sehgal M. Song C. Ehlers V. L. Moyer J. R. Jr. (2013). Learning to learn—intrinsic plasticity as a metaplasticity mechanism for memory formation. Neurobiol. Learn. Mem. 105, 186–199 10.1016/j.nlm.2013.07.008 23871744 PMC3855019

[B87] Selverston A. I. Russell D. F. Miller J. P. (1976). The stomatogastric nervous system: structure and function of a small neural network. Prog. Neurobiol. 7, 215–290 10.1016/0301-0082(76)90008-3 11525

[B88] Shabb J. B. (2011). “Cyclic nucleotide specificity and cross-activation of cyclic nucleotide receptors,” inTransduction Mechanisms in Cellular Signaling, eds Dennis E. A. Bradshaw R. A. (Oxford, UK: Academic Press), 441–446

[B89] Smith W. B. Starck S. R. Roberts R. W. Schuman E. M. (2005). Dopaminergic stimulation of local protein synthesis enhances surface expression of GluR1 and synaptic transmission in hippocampal neurons. Neuron 45, 765–779 10.1016/j.neuron.2005.01.015 15748851

[B90] Sosanya N. M. Huang P. P. Cacheaux L. P. Chen C. J. Nguyen K. Perrone-Bizzozero N. I. (2013). Degradation of high affinity HuD targets releases Kv1.1 mRNA from miR-129 repression by mTORC1. J. Cell. Biol. 202, 53–69 10.1083/jcb.201212089 23836929 PMC3704988

[B91] Steinberg E. E. Keiflin R. Boivin J. R. Witten I. B. Deisseroth K. Janak P. H. (2013). A causal link between prediction errors, dopamine neurons and learning. Nat. Neurosci. 16, 966–973 10.1038/nn.3413 23708143 PMC3705924

[B92] Tan X. Wang S. Yang B. Zhu L. Yin B. Chao T. (2012a). The CREB-miR-9 negative feedback minicircuitry coordinates the migration and proliferation of glioma cells. PLoS One 7:e49570 10.1371/journal.pone.0049570 23185366 PMC3502497

[B93] Tan X. Wang S. Zhu L. Wu C. Yin B. Zhao J. (2012b). cAMP response element-binding protein promotes gliomagenesis by modulating the expression of oncogenic microRNA-23a. Proc. Natl. Acad. Sci. U S A 109, 15805–15810 10.1073/pnas.1207787109 23019365 PMC3465427

[B94] Temporal S. Desai M. Khorkova O. Varghese G. Dai A. Schulz D. J. (2012). Neuromodulation independently determines correlated channel expression and conductance levels in motor neurons of the stomatogastric ganglion. J. Neurophysiol. 107, 718–727 10.1152/jn.00622.2011 21994267 PMC3349629

[B95] Thoby-Brisson M. Simmers J. (1998). Neuromodulatory inputs maintain expression of a lobster motor pattern-generating network in a modulation-dependent state: evidence from long-term decentralization in vitro. J. Neurosci. 18, 2212–2225 9482805 10.1523/JNEUROSCI.18-06-02212.1998 PMC6792931

[B96] Thoby-Brisson M. Simmers J. (2002). Long-term neuromodulatory regulation of a motor pattern-generating network: maintenance of synaptic efficacy and oscillatory properties. J. Neurophysiol. 88, 2942–2953 10.1152/jn.00482.2001 12466420

[B97] Till S. Lejeune E. Thermann R. Bortfeld M. Hothorn M. Enderle D. (2007). A conserved motif in Argonaute-interacting proteins mediates functional interactions through the Argonaute PIWI domain. Nat. Struct. Mol. Biol. 14, 897–903 10.1038/nsmb1302 17891150

[B98] Trantham-Davidson H. Neely L. C. Lavin A. Seamans J. K. (2004). Mechanisms underlying differential D1 versus D2 dopamine receptor regulation of inhibition in prefrontal cortex. J. Neurosci. 24, 10652–10659 10.1523/jneurosci.3179-04.2004 15564581 PMC5509068

[B99] Tsai N. P. Lin Y. L. Wei L. N. (2009). MicroRNA mir-346 targets the 5′-untranslated region of receptor-interacting protein 140 (RIP140) mRNA and up-regulates its protein expression. Biochem. J. 424, 411–418 10.1042/BJ20090915 19780716

[B100] Vasudevan S. Tong Y. Steitz J. A. (2007). Switching from repression to activation: microRNAs can up-regulate translation. Science 318, 1931–1934 10.1126/science.1149460 18048652

[B101] Vo N. Klein M. E. Varlamova O. Keller D. M. Yamamoto T. Goodman R. H. (2005). A cAMP-response element binding protein-induced microRNA regulates neuronal morphogenesis. Proc. Natl. Acad. Sci. U S A 102, 16426–16431 10.1073/pnas.0508448102 16260724 PMC1283476

[B102] Wall V. Z. Parker J. G. Fadok J. P. Darvas M. Zweifel L. Palmiter R. D. (2011). A behavioral genetics approach to understanding D1 receptor involvement in phasic dopamine signaling. Mol. Cell. Neurosci. 46, 21–31 10.1016/j.mcn.2010.09.011 20888914 PMC3035386

[B103] Wang D. O. Kim S. M. Zhao Y. Hwang H. Miura S. K. Sossin W. S. (2009). Synapse- and stimulus-specific local translation during long-term neuronal plasticity. Science 324, 1536–1540 10.1126/science.1173205 19443737 PMC2821090

[B104] Wen W. Taylor S. S. (1994). High affinity binding of the heat-stable protein kinase inhibitor to the catalytic subunit of cAMP-dependent protein kinase is selectively abolished by mutation of Arg133. J. Biol. Chem. 269, 8423–8430 8132568

[B105] Wibrand K. Panja D. Tiron A. Ofte M. L. Skaftnesmo K. O. Lee C. S. (2010). Differential regulation of mature and precursor microRNA expression by NMDA and metabotropic glutamate receptor activation during LTP in the adult dentate gyrus in vivo. Eur. J. Neurosci. 31, 636–645 10.1111/j.1460-9568.2010.07112.x 20384810 PMC3791877

[B106] Yanow S. K. Manseau F. Hislop J. Castellucci V. F. Sossin W. S. (1998). Biochemical pathways by which serotonin regulates translation in the nervous system of Aplysia. J. Neurochem. 70, 572–583 10.1046/j.1471-4159.1998.70020572.x 9453551

[B107] Yuan S. Burrell B. D. (2013). Endocannabinoid-dependent long-term depression in a nociceptive synapse requires coordinated presynaptic and postsynaptic transcription and translation. J. Neurosci. 33, 4349–4358 10.1523/JNEUROSCI.3922-12.2013 23467351 PMC3640288

[B108] Zhang Y. MacLean J. N. An W. F. Lanning C. C. Harris-Warrick R. M. (2003). KChIP1 and frequenin modify shal-evoked potassium currents in pyloric neurons in the lobster stomatogastric ganglion. J. Neurophysiol. 89, 1902–1909 10.1152/jn.00837.2002 12612050

[B109] Zhang H. Rodgers E. W. Krenz W. D. Clark M. C. Baro D. J. (2010). Cell specific dopamine modulation of the transient potassium current in the pyloric network by the canonical D1 receptor signal transduction cascade. J. Neurophysiol. 104, 873–884 10.1152/jn.00195.2010 20519576 PMC2934939

[B110] Zoli M. Torri C. Ferrari R. Jansson A. Zini I. Fuxe K. (1998). The emergence of the volume transmission concept. Brain Res. Brain Res. Rev. 26, 136–147 10.1016/S0165-0173(97)00048-9 9651506

[B111] Zuo P. L. Yao W. Sun L. Kuo S. T. Li Q. Wang S. R. (2013). Impulse-dependent extracellular resting dopamine concentration in rat striatum in vivo. Neurochem. Int. 62, 50–57 10.1016/j.neuint.2012.11.006 23159778

